# Outcomes of Minimally Invasive Adrenalectomy for Large Adrenal Masses: A Multi-Centre Experience in Saudi Arabia

**DOI:** 10.7759/cureus.55276

**Published:** 2024-02-29

**Authors:** Raed A. Azhar, Omar Buksh, Abdullah M Almalki, Rabea Akram, Hani Alzahrani, Abdullah Al-Gadheeb, Qusay Mandoorah, Adel A Alammari

**Affiliations:** 1 Urology Department, King Abdulaziz University, Faculty of Medicine, Jeddah, SAU; 2 Urology Department, International Medical Center, Jeddah, SAU; 3 Urology Department, King Faisal Specialist Hospital and Research Centre, Jeddah, SAU; 4 Urology, Prince Sultan Military Medical City, Riyadh, SAU

**Keywords:** robotic adrenalectomy, laparoscopic adrenalectomy, minimally invasive adrenalectomy, urology, large adrenal mass, adrenalectomy

## Abstract

Introduction

Advancements in radiological imaging technology have increased the discovery of adrenal incidentalomas. Large adrenal tumors (LATs) are not common, and the likelihood of malignancy increases with tumor size. LATs were defined as tumors larger than four centimeters (cm) with various pathologic diagnoses. Traditionally, open adrenalectomy was considered the gold standard for LATs, but with recent advancements in minimally invasive surgery (MIS), optimum perioperative and long-term outcomes are achievable by the MIS approach._ _The findings presented in this paper show that even large adrenal masses measuring up to 21 centimeters can be safely removed using a minimally invasive approach.

Methodology

After Institutional Review Board (IRB) approval, we reviewed medical records of adult patients who had adrenalectomies at two Saudi Arabian centers from January 2013 to February 2023. Inclusion criteria were laparoscopic or robotic adrenalectomy and adrenal lesions ≥5cm. Pediatric patients and those with open adrenalectomies were excluded. Pre-surgery, patients had imaging studies to assess mass characteristics. Pheochromocytoma patients received a 2-week adrenergic blocker treatment. Perioperative data including demographics, comorbidities, mass characteristics, surgery details, and follow-up were analyzed using SPSS-23. Patients provided informed consent and had follow-up appointments and imaging.

Results

Our experience involved 35 patients, 29 of whom received laparoscopic treatment and six of whom underwent robotic surgery. Of the 35 patients, more than half were females (57.1%), with a mean age of 41.7±14.9 years, the youngest and oldest participants being 16 and 73 years of age, respectively. The mean body mass index (BMI) of the participants was in the overweight range (26±6.0 kg/m^2^). The most common mode of presentation was incidental (42.9%), followed by hypertension (17.1%). Most patients had right-sided adrenal gland involvement (48.6%), with only four patients showing bilateral involvement. Most of the patients were classified as American Society of Anesthesiology score (ASA) 2 (40.0%) or ASA 3 (40.0%). Most of the patients were diagnosed with myelolipoma or adenoma (22.9% each) followed by pheochromocytoma (17.1%). The average estimated blood loss (EBL) was 189.3±354.6 ml for patients who underwent laparoscopic surgery and 80.0 ±34.6 ml for patients who underwent robotic surgery. The average operative room time (ORT) was 220.1±98.7 minutes (min) for laparoscopic surgery and 188.3±10.3 min for robotic surgery. One patient had to be converted from laparoscopic to open surgery due to aortic injury. The average length of stay (LOS) was 9.5±6.7 days for laparoscopic treatment and 5.5±1.9 days for robotic surgery. The mean tumor size in the greatest dimension was 8.0±4.4 cm. Only one patient who underwent unilateral laparoscopy experienced perioperative complications and converted to open surgery; nine patients who underwent unilateral laparoscopy required blood transfusion, and none of the patients who underwent robotic surgery required transfusion. None of the 35 patients experienced a recurrence of their adrenal disease during the mean follow-up period which lasted around 58 months.

Conclusion

MIS in Saudi Arabia is growing and is a safe method for LATs, with satisfactory surgical results compared to the traditional open surgery approach. It offers advantages in terms of EBL, complications, and disease recurrence.

## Introduction

Due to recent advancements in imaging technology, there has been an increase in the discovery of adrenal incidentalomas. However, large adrenal tumors (LATs) are not common, the incidence of which ranges from 8.6% to 38.6% [1-2]. The likelihood of malignancy increases with increasing tumor size [3-4]. It is crucial to accurately assess the nature and origin of LATs before surgery to determine the appropriate surgical approach [5-6]. LATs are defined as tumors >5-10cm in diameter, with a consensus of ~5cm [7-8].

Ever since Gagner et al. first introduced the concept in 1991, the minimally invasive surgery (MIS) approach for treating various adrenal lesions has gained significant popularity [9]. In comparison to the traditional open surgical technique, laparoscopic adrenalectomy has been shown to have reduced perioperative complications and mortality rates, along with shorter hospital stays and improved cosmetic outcomes. This has led to an increasing preference for the MIS approach among surgeons and patients alike.

Typically, due to concerns about incomplete resection and the possibility of local invasion, an open surgical approach is recommended for LATs [10]. In addition, the MIS approach for large adrenal masses has not gained worldwide acceptance for adrenalectomy due to its drawbacks, such as higher cost and limited accessibility. Nevertheless, the current standard of care favors an open approach for tumors larger than five cm, as minimally invasive procedures are considered contraindicated in such cases. Gryn et al. found that tumors >5cm in size are associated with unfavorable surgical outcomes, positive margins, and complications [10]. However, with rapid improvements in MIS techniques such as robotic and laparoscopic approaches, LATs are being increasingly resected using MIS, with acceptable and safe outcomes [8].

In this paper, we demonstrate our experience with using MIS as a surgical approach for large adrenal masses, and that even LATs reaching 21 cm can be safely resected with the MIS approach with acceptable blood loss, length of stay (LOS), operative room time (ORT), and optimum oncological outcomes.

## Materials and methods

Following the approval from the Institutional Review Board (IRB) (Approval number: 2021-02), we conducted a retrospective review of the medical records of adult patients who underwent adrenalectomies at two tertiary centers in Saudi Arabia (in King Faisal Specialist Hospital and Research Centre Jeddah and King Abdulaziz University Hospital Jeddah) between the period January 1, 2013, and February 30, 2023. Our inclusion criteria consisted of adult patients over the age of 18, who underwent laparoscopic or robotic adrenalectomy, for adrenal lesions measuring five centimeters or more. Pediatric patients and those who underwent open adrenalectomies were excluded from the study. Thirty-five patients met the inclusion criteria.

Prior to surgery, all patients underwent various imaging studies, including ultrasound, abdominal cross-sectional (CT) scan, magnetic resonance imaging (MRI), and metaiodobenzylguanidine (MIBG) scintigraphy for suspected adrenal pheochromocytoma, to assess the location, size, and functional characteristics of the mass. Functional masses were identified through serum levels of aldosterone and cortisol, as well as urinary levels of metanephrines. Patients diagnosed with pheochromocytoma received a two-week treatment with oral adrenergic blockers, and intraoperative monitoring was performed to ensure hemodynamic stability. We thoroughly examined the perioperative data of the patients, which encompassed factors such as age, gender, body mass index (BMI), comorbidities, smoking status, history of malignancy, presentation of the mass, functionality of the mass, size and laterality of the mass, pathology of the mass, modality of surgery, operative time, blood loss and if blood transfusion was required intra-operatively or postoperatively, intra-operative complications, length of hospital stay, duration of follow-up, and recurrence rates. The data was collected and then analyzed using SPSS-23 (IBM SPSS Statistics for Windows, Version 23.0. Armonk, NY: IBM Corp.), with a p-value of less than 0.05 considered statistically significant. All patients provided informed consent for the surgeries after a comprehensive explanation of the associated risks and benefits. Follow-up appointments were scheduled for patients diagnosed with malignant disease at 3, 6, and 12 months post-surgery, during which cross-sectional (CT) scans were conducted. Annual CT scans of the chest, abdomen, and pelvis were done afterward yearly for a presumed follow-up period of 60 months.

## Results

The final study included a total of 35 patients, out of which 29 underwent laparoscopic surgery while six underwent robotic surgery. The mean age of those who underwent laparoscopic surgery was higher than those who underwent robotic surgery (p=0.5). The majority of laparoscopic cases were females, while the gender ratio was equal in robotic cases. The body mass index (BMI) of laparoscopic cases was higher but without statistical significance. None of the patients in the robotic surgery group were diabetic. The operation room time (ORT), estimated blood loss (EBL), and length of stay in the hospital (LOS) were all noted to be higher for laparoscopic cases than robotic cases but without statistical significance. Mean tumor size was higher in the robotic technique, with a mean size of 8.9± 6.2 cm but without statistical significance, with the largest tumor size of 21 cm. Details of the baseline demographics have been depicted in (Table 1).

**Table 1:** Details of the baseline demographics

Variable	Value
Sex: (n) (%):	
Males	Males = 15 (42.5%)
Females	Females = 20 (57.1%)
Mean age in years ± Standard deviation	41.7 ± 14.9
Mean weight in kilograms ± Standard deviation	88.1 ± 40.5
Mean height in centimeters ± Standard deviation	148.1 ± 34.2
Mean body mass index in kilograms/m^2^ ± Standard deviation	26.9 ± 6
Mean tumor size in centimeters ± Standard deviation	8 ± 4.6
Comorbidity (n) (%):	
Hypertension	15 (42.9%)
Diabetes	4 (11.4%)
Smokers (n) (%)	0 (0%)
Past history of malignancy (n) (%)	5 (12.5%)
History of cardiovascular disease (n) (%)	2 (5%)
Hypertensive/hypotensive crises	0
Presentation (n) (%):	
Incidental	15 (42.9%)
Hypertension	6 (17.1%)
Adrenal gland disorder	4 (11.4%)
Flank pain	4 (11.4%)
Palpitation	3 (6.6%)
Other	3 (8.6%)
Involvement (n) (%):	
Left	14 (40%)
Right	17 (48.6%)
Bilateral	4 (11.4%)
Endocrine Functioning status (n) (%):	
Functional	13 (37.1%)
Nonfunctional	22 (62.9%)
Procedure (n) (%):	
Unilateral laparoscopy	25 (62.5%)
Bilateral laparoscopy	4 (10%)
Robotic	6 (15%)
Final diagnosis (n) (%):	
Myolipoma	8 (22.9%)
Adenoma	8 (22.9%)
Pheochromocytoma	6 (17.1%)
Metastatic lesion	3 (8.6%)
Cyst	2 (5.7%)
Benign lesion	3 (8.6%)
Other	5 (14.3%)

Analysis of the means of the operative variables revealed that robotic procedures had the shortest time in the operation room (ORT) at 183.3±10.3 minutes, while bilateral laparoscopic procedures had the longest at 321±64 minutes. This difference in ORT was statistically significant (p=0.03). Unilateral laparoscopic surgeries had the highest mean blood loss at 214±386.3 ml, while both robotic and bilateral laparoscopic procedures had a lower mean blood loss. Bilateral laparoscopies had a slightly lower mean blood loss (76.3±33 ml) compared to robotic surgeries (80±34.6 ml), but this difference was not statistically significant (p=0.58). Robotic surgeries had the shortest mean hospital stay at 5.5±1.9 days, while unilateral laparoscopic surgeries had the longest mean hospital stay at 9.4±6.3 days. However, these differences were not statistically significant (p=0.25). Details of the comparison drawn between the operative and post-operative variables have been depicted in (Table 2).

**Table 2:** A comparison of the means of operative and post-operative variables based on surgical technique using the one-way ANOVA test P-value < 0.05 is considered as significant.

Variable	Unilateral Lap	Bilateral Lap	Robotic	p-value
Operative room time in minutes ± Standard deviation	202.3 ± 96.0	321 ± 64	183.3 ± 10.3	0.03
Estimated blood loss in milliliters ± Standard deviation	214 ± 386.3	76.3 ± 33	80 ± 34.6	0.58
Length of stay in days ± Standard deviation	9.4 ± 6.3	6.5 ± 3.8	5.5 ± 1.9	0.21

In terms of complications, the overall recurrence rate was low, with a peri-operative complication being reported in two cases only, which was in the unilateral laparoscopy group. A similar trend was noted in the same arm for conversion to open surgery due to aortic injury. With regards to cases needing blood transfusions, nine cases of unilateral laparoscopy required blood transfusions. The mean follow up period was 58 months, with a range from one to five years, and none of the 35 patients included in the study experienced any recurrence of their adrenal disease. When the data was sub-grouped and analyzed based on tumor size greater than or less than seven cm in the greatest dimension, results showed that all parameters (mean ORT, EBL, and LOS) were lower for tumor size <7 cm but without statistical significance. Further details on the data are in (Table 3).

**Table 3:** A comparison of the means of operative and post-operative variables based on tumor size using the One-way ANOVA test P-value < 0.05 is considered as significant.

Variable	Tumor size < 7 cm	Tumor size > 7 cm	p-value
Operative room time in minutes ± Standard deviation	197.5 ± 79.3	241.3 ± 105.1	0.17
Estimated blood loss in milliliters ± Standard deviation	130.9 ± 238.1	238.1 ± 430.5	0.35
Length of stay in days ± Standard deviation	8.1 ± 6.1	10.1 ± 6.7	0.38

When the data was sub-grouped and analyzed based on site of tumor, results showed that ORT was the lowest for right-sided tumors and highest for bilateral tumors; these findings were statistically significant (p=0.03). Interestingly, EBL was highest for right sided tumors and lowest for bilateral tumors but without statistical significance. Another peculiar finding was that LOS was lowest for bilateral tumors and highest for left sided tumors, but again without statistical significance. Further details are demonstrated in Table 4.

**Table 4:** A comparison of the means of operative and post-operative variables based on laterality of tumor using the One-way ANOVA test P-value < 0.05 is considered as significant.

Variable	Left	Right	Bilateral	p-value
Operative room time in minutes ± Standard deviation	202.2 ± 81	198 ± 90.4	321 ± 64.0	0.03
Estimated blood loss in millileters ± Standard deviation	98.9 ± 131.6	251.8 ± 442.9	76.3 ± 33	0.36
Length of stay in days ± Standard deviation	10.6 ± 8.2	7.9 ± 4.7	6.5 ± 3.9	0.37

## Discussion

Adrenal tumors are frequently discovered incidentally during abdominal radiology scans, particularly in older individuals [11]. The size of the tumor can serve as an indicator of its potential malignancy, with larger tumors having a higher likelihood of being cancerous. However, linking the size with the likelihood of malignancy is not always simple. Numerous studies involving a significant number of patients with adrenal tumors have demonstrated that around 10% or less of these cases are malignant [12,13]. As a result, opting for an open surgical approach for large adrenal tumors and reserving minimally invasive surgery for smaller masses may not always be the optimal choice.

From our perspective, it is crucial to possess expertise in laparoscopic adrenal surgery and proficiency in robotic procedures to effectively avoid complications. Various studies have documented that the reasons for converting from robotic surgery to laparoscopic or open adrenalectomy include visceral injury, challenges in achieving hemostasis, incorrect placement of robotic trocars, and prolonged duration of the operation [14]. These factors highlight the significance of having experience and skill in both laparoscopic and robotic techniques to minimize the likelihood of encountering such complications. In our study, only one case converted from laparoscopic to open surgery, which was due to aortic injury.

A literature review examined the use of minimally invasive surgery (MIS) for adrenalectomies, and six studies were analyzed [15-20] (Table 5). Four studies used laparoscopy exclusively [15-18], one used both laparoscopy and robotic techniques [19], and one used robotic surgery exclusively [20]. The number of patients in each study varied from 10 to 52, with an average age range of 42.8 to 57 years. Most studies had a similar male-to-female ratio. Tumor laterality differed among the studies. The average BMI ranged from 24.1 Kg/m2 to 31.1 Kg/m2, and the mean tumor size ranged from 5.94cm to 8cm. EBL during surgery ranged from 54.4ml to 400ml, and OR time ranged from 89.6 minutes to 205 minutes. Across all studies, eleven patients required conversion to open surgery, and LOS in the hospital ranged from 1.4 days to 4.1 days. A quick comparison between our results with the data above shows that the data obtained from previous studies aligns closely with our results, suggesting that the MIS approach can be considered a safe method for resecting LATs. The similarity between the two sets of data highlights the reliability and consistency of our findings, further supporting the notion that MIS can be a viable option for the resection of LATs.

**Table 5:** A literature review of similar cases that was published between 2000-2020

Author	Year of publication	Surgical technique	Number of patients	Mean age in years ± Standard deviation	Gender (Male/Female) (n)	Laterality (Right/Left/Bilateral) (n)	Mean Body mass index in Kg/m2 ± Standard deviation	Mean size in centimeters ± Standard deviation	Estimated blood loss in milliliters ± Standard deviation	Operation room time in minutes ± Standard deviation	Conversion to open (n)	Length of stay in days ± Standard deviation
Hobart et al., [15]	2000	Laparoscopy	14	57.1	6/8	8/6/0	31.4	8	400	205	2	2.4
Henry et al., [16]	2002	laparoscopy	19	57.3	4/15	10/9/0	Not mentioned	7.1	Not mentioned	150	2	Not mentioned
Naya et al., [17]	2005	Laparoscopy	16	48.1 ± 15.6	12/4	5/11/0	24.1 ± 3.2	Not mentioned	212 ± 165	175 ± 82	0	Not mentioned
Bhat et al., [18]	2007	Laparoscopy	10	42.8 ± 10.65	5/5	4/5/1	Not mentioned	8 cm ± 1.47	116.5	157	2	4.1
Agcaoglu et al., [19]	2012	Laparoscopy	38	52.5 ± 2.3	18/20	13/25/0	30.2 ± 0.9	6.2 ± 0.3	166.6 ± 51.2	187.2 ± 8.3	4	1.9 ± 0.1
		Robotic surgery	24	52.4 ± 2.9	10/14	9/16/0	27.1 ± 0.8	6.5 ± 0.4	83.6 ± 59.4	159.4 (± 13.4)	1	1.4 ± 0.2
Ragavan et al., [20]	2020	Robotic surgery	38	46.5 ± 11.3	21/17	22/16/0	Not mentioned	5.94 ± 3.2	54.4 ± 16.8	89.6 ± 8.1	0	2.7 ± 2.0
Our study	2023	Laparoscopy	29	42.5 ± 16.2	12/17	12/13/4	27.5 ± 6.3	7.8 ± 4.4	189.3 ± 354.6	220.1 ± 98.7	1	9.5 ± 6.7
		Robotic Surgery	6	38.0 ± 3.5	3/3	5/1	24.0 ± 2.4	8.9 ± 6.2	80.0 ± 34.6	188.3 ± 10.3	0	5.5 ± 1.9

**Figure 1:**
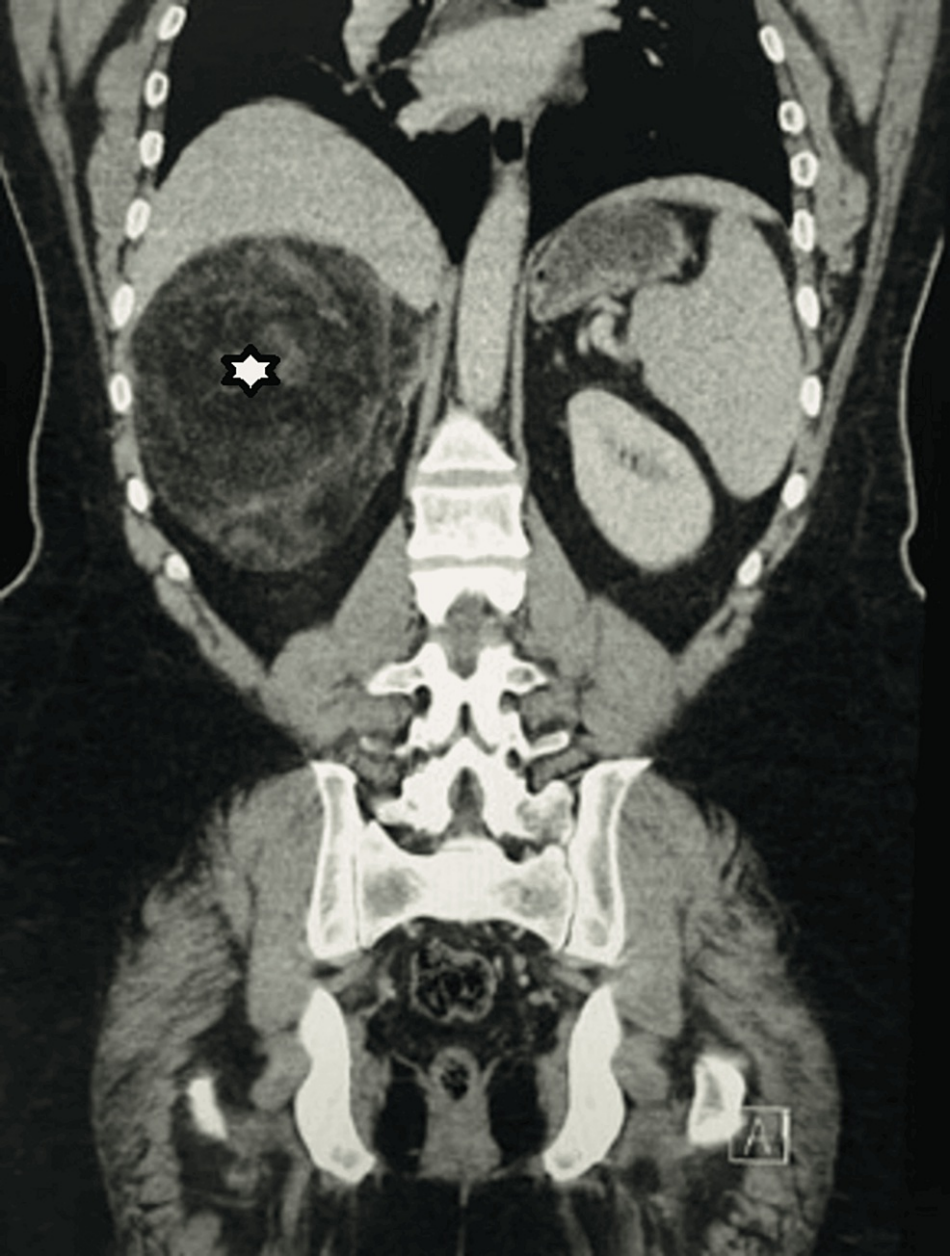
CT (Computed Tomography) image showing the largest mass in our study.

**Figure 2:**
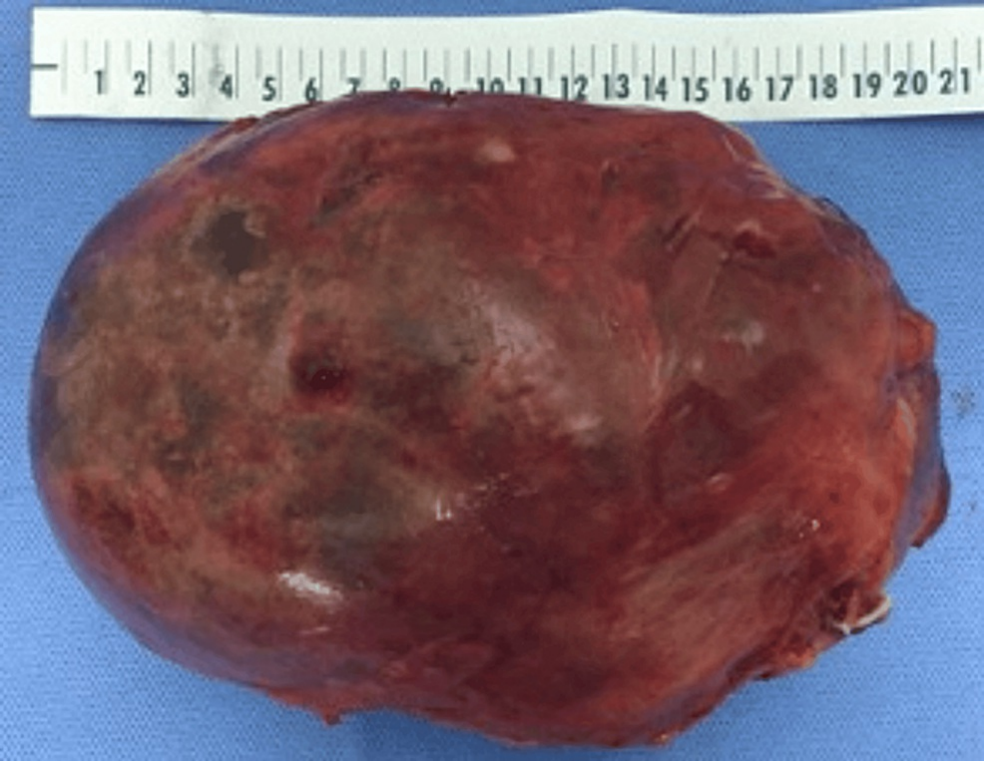
Postoperative image of the largest adrenal mass in our study, a 21 cm adrenal myelolipoma that was successfully resected using a robotic approach.

A variety of pathological types are observed in LATs. Therefore, when diagnosing LATs clinically, it is important to consider the possibility of benign tumors first. However, there is still a significant chance that a LAT could be malignant. Previous research found that benign LATs made up 62.59% while malignant LATs made up 37.41% of the total LATs [1-2]. Our study showed that 8.57% of the 35 cases included were malignant lesions. As for the pathology results, reports of the resected LATs in our study demonstrate that myelolipoma and adenoma were the two most common entities, with each occurring in 22.9% of patients in our study. Interestingly, the largest mass excised was an adrenal myelolipoma that was 21 cm in size (Figures 1,2); the mass was excised using a robot, and no complications occurred, nor was any blood transfusion required.

During adrenal surgery, the size of the tumor is an important factor to consider as the adrenal capsule could potentially rupture if great care is not taken. Tumors larger than five cm with a ruptured capsule pose an oncological risk during surgery. Laparoscopic equipment has limitations in movement and access to interior parts of the abdomen, which increases the risk of capsular rupture in large tumors and obese patients. The authors believe that robotic technology can handle tumors efficiently without causing capsular rupture, and can achieve the principle of controlling the adrenal vein as a first step in surgery. One author in the study successfully excised a 21 cm giant adrenal myolipoma using robotic technology (Figure 1,2). Comparative studies have shown that robotic approaches have better outcomes than laparoscopic approaches, with shorter operative times, lower morbidity rates, and fewer conversions [21-23]. Our research involved comparing the outcomes of unilateral laparoscopy, bilateral laparoscopy, and robotic laparoscopy in terms of EBL, ORT, and LOS. The use of robotic surgery resulted in lower rates of ORT and EBL as well as a shorter hospital. However, the availability of the robot in addition to the training of the surgeon limits its use.

Regardless of the size, adrenalectomy can be challenging based on its laterality. Right-sided adrenalectomies are complicated by the retrocaval location and short adrenal vein, while left-sided adrenalectomies require more mobilization and proximity to the pancreas. Previous research has shown that right adrenalectomies have shorter operative time and less blood loss than left adrenalectomies [24]. In our study, we found that a right adrenalectomy had a shorter operative time than a left or bilateral adrenalectomy. Unexpectedly, bilateral adrenalectomies had less blood loss and a shorter hospital stay compared to left or right adrenalectomy.

Although our study is the first in Saudi Arabia and the Middle East to focus on MIS for LATs, it is limited by several factors. First, the small sample of patients limits the ability to draw any strong conclusions. Additionally, the retrospective nature of the study adds to its limitations. However, we hope that this study encourages urologists in Saudi Arabia and the Middle East to continue to perform robotic and laparoscopic MIS for LATs, thus allowing us to perform a proper clinical trial for adequate comparison in the future.

## Conclusions

With MIS being increasingly performed in Saudi Arabia, MIS for LATs is considered a safe approach that yields adequate surgical outcomes when compared to the traditional open approach in terms of EBL, perioperative complications, and more importantly, disease recurrence during the follow-up period. We hope that this study, within its limitations, encourages young skilled MIS urologists in the region to perform adrenalectomies by an MIS approach, rather than immediately resorting to an open surgical approach for LATs.
